# Associations between cognitive function and lifestyle in community-living older people: a correlational study

**DOI:** 10.1186/s13104-024-06766-z

**Published:** 2024-04-09

**Authors:** Maryam Zarringhadam, Shirin Hasanvand, Mehdi Birjandi, Afsaneh Beiranvand

**Affiliations:** 1 Student Research Committee, Lorestan University of Medical Sciences, Khorramabad, Iran; 2 Social Determinants of Health Research Center, School of Nursing & Midwifery, Lorestan University of Medical Sciences, Khorramabad, Iran; 3 Nutritional Health Research Center, School of Health and Nutrition, Lorestan University of Medical Sciences, Khorramabad, Iran

**Keywords:** Cognitive function, Lifestyle factors, Older adults, Cognitive disorder

## Abstract

**Background:**

Previous studies have examined relationship between cognitive function and lifestyle; however, the nature of this relationship is expected to vary in diverse cultural and low-income setting where lifestyle practices differ from those in high-income countries.

**Aim:**

This study aims to investigate the correlation between lifestyle factors and cognitive function among individuals aged 60 years and older in 2021.

**Methods:**

This cross-sectional, Community-based study involved 300 older adults from comprehensive urban health centers in Khorramabad, Iran, selected through stratified cluster sampling. Data were collected using the demographic information questionnaire, Mini-Mental State Examination, and Lifestyle Questionnaire. Data management and analysis were performed using SPSS (version 22) and independent t-tests, Pearson's correlation coefficient, ANOVA, and multiple linear regression analysis were used. A p value < 0.05 was considered significant.

**Results:**

The study included 156 males (52%) and 144 females (48%). Findings revealed a significant correlation between cognitive function and lifestyle (*P* < *0.001*). Multiple linear regression analysis indicated that physical health, environmental health, exercise, accident prevention, and avoidance of medication exerted the most significant positive effect on cognitive function. Conversely, social health exhibited a notable negative influence on cognitive function. (*P* < *0.001*).

**Conclusion:**

The results suggest that specific aspects of lifestyle, such as physical health, accident prevention, and avoidance of medication are associated with cognitive function in older adults. Consequently, lifestyle promotion programs may enhance cognitive function and improve the quality of life among older adults.

## Introduction

Population aging is a major challenge for public health in developing countries [1]. The increase in number of older adults is expected to lead to an increase in the prevalence of age-related diseases, including cognitive impairment [2, 3]. The estimated global prevalence of dementia is as high as 50 million [4]. Estimates in Asia vary somewhat, possibly due to diverse diagnostic criteria and variations in assessment tools. [5]. The older population of Iran is rapidly increasing due to the rapid decrease in fertility rate and increase in life expectancy [6]. It is predicted that the percentage of people aged over 65 will rise from 5.7% in 2011 to 9.7% in 2030 and to 25.2% in 2060 [7] cognitive disorders pose an elevated risk of disability, leading to a substantial decline in the quality of life. Moreover, the high cost of the disease imposes a considerable economic burden on families and the country's health system [8]. The estimated global cost in 2015 reached 818 million dollars, comprising approximately 1.09% of the world’s aggregated Gross Domestic Product. According to the World Alzheimer Association's projection, the number of individuals with Alzheimer's disease reach about 131 million by 2050 [9], with the majority of the increase occurring in developing countries [7]. Since cognitive decline is mainly irreversible, it is crucial Preventing or delaying cognitive decline. Norton and colleagues conducted a meta-analysis and demonstrated that approximately one-third of Alzheimer's disease cases can be attributed to modifiable risk factors, which could prevent a significant number of dementia cases in the future [10]. Lifestyle is an important modifiable factor influencing cognitive function, encompassing nutrition, physical activity, stress, smoking, and sleep quality [11, 12]. A study by Clare et al. (2017) revealed that a healthy lifestyle, involving cognitive and social activities, physical activity, and a healthy diet, positively influences cognitive function. Thus, cognitive health is maintained with a healthy and active lifestyle [13]. Although older adults in low- and middle-income countries (LMICs) may be particularly vulnerable to effects of cognitive impairment and dementia, population-based studies of cognitive aging in these regions remain rare [14].Despite the importance of this topic, there is a paucity of studies on the cognitive function of older adults in Iran, as indicated by the literature review. Only two studies have focused on retired armed forces personnel and older adults residing in nursing homes [15, 16]. Therefore, it is imperative to examine the cognitive function of older adults at the community level, encompassing a broader spectrum of older individuals rather than concentrating solely on specific groups. Furthermore, the World Health Organization asserts that nearly 60% of individuals' quality of life and health depends on their lifestyle. Additionally, statistics on the leading causes of death reveal that 53% of deaths are attributed to people's lifestyles and unhealthy behaviors [17]. Acknowledging the pivotal role of lifestyle in preventing cognitive disorders in older adults, the current study aims to investigate the relationship between cognitive function and the lifestyle of older individuals.

## Materials and methods

### Study design and setting

This study is a cross-sectional, Community-based survey, conducted from August 2021 to April 2022 in Khorramabad city, in western Iran. Participants were recruited from comprehensive urban health centers.

### Participants and sampling

A total of 300 eligible older adults participated. Based on stratified cluster sampling, Khorramabad city was divided into three parts: north, center, and south, each treated as a stratum. Each comprehensive urban health service center was considered a cluster (n = 15), then seven clusters were randomly selected out of the three stratum. The list of older adults covered by the comprehensive urban health service centers was obtained. After completing administrative procedures and reviewing legal documents from the centers, participants were invited using a phone call explaining the research objectives. The inclusion criteria included age 65 or older, literacy in reading and writing, proficiency in Persian and local dialects, absence of severe vision and hearing disorders, and no recent surgery or local anesthesia use in the last month. Criteria for excluding the subjects were as follows: a history of neurological and psychiatric disorders like Parkinson's disease or epilepsy, alcoholism, severe head trauma, significant diseases such as cardiac failure, ongoing cancer treatment, severe hepatic or renal dysfunction, and mobility difficulties due to stroke. Older adults who submitted incomplete questionnaires or chose not to continue participating were excluded.

### Data collection

Participants aged 60 years or older were invited to comprehensive health centers, where the study's objectives were explained. Consent was obtained from those willing to participate. The enrollment commenced on August 1, 2021, and lasted approximately six months.

Data collection tools included Sociodemographic questionnaires, Mini-Mental State Examination (MMSE) and Lifestyle Questionnaire (LSQ which were completed by the participants.

### Assessment of sociodemographic characteristics

Sociodemographic characteristics included age, gender, marital status, education level, BMI, income status, insurance status, cohabitation status, driving, and underlying diseases, was collected. Moreover, at baseline, interviews were conducted to evaluate clinical history including underlying disease and history of cardiovascular risk factors (hypertension, diabetes mellitus, and hypercholesterolemia). BMI was calculated by measuring weight (kg) and height (m) and then dividing weight by the square of height (m^2^). The World Health Organization -Europe classification was used for BMI: < 18.5 kg/m^2^ as underweight, 18.5–24.4 kg/m^2^ as normal weight, 25–29.9 kg/m^2^ as overweight, and ≥ 30 kg/m^2^ as obesity [18].

### Assessment of lifestyle factors

The lifestyle was assessed using the Lifestyle Questionnaire (LSQ) with 10 components including physical health, exercise and wellness, weight control and nutrition, disease prevention, psychological health, spiritual health, social health, avoidance of medication and alcohol, accident prevention, and environmental health. Utilizing a 4-point Likert scale ranging from never (0) to always (3), the questionnaire yields a total score ranging from 0 to 198, with a higher score indicating a more favorable lifestyle. A score falling within 0–70 signifies an undesirable lifestyle, 70–105 denotes an average lifestyle, and scores exceeding 105 indicate a desired lifestyle. Originating from Iran, Laali et al. (2012) designed this tool, with reliability confirmed through internal consistency method with Cronbach's alpha ranging between 0.76 and 0.89 [19]. In the present study, the reliability was assessed through internal consistency, with lifestyle components demonstrating α values of 0.72–0.97 and an overall α of 0.95.

### Assessment of cognitive function

Cognitive function was assessed using Persian versions of the Mini-Mental State Examination. This scale designed by Folstein (1975) to screen dementia [20] and evaluate various cognitive domains: orientation (10 questions), registration (3 questions), attention-calculation (5 questions), recent memory (3 questions), different language functions (3 questions) and visual-spatial thinking (6 questions).With 30 questions in total, subjects' scores range from 0 to 30. Scores below 9 indicate severe cognitive dysfunction, 10–20 denote moderate dysfunction, 21–24 signify mild dysfunction, and 25 and above indicate cognitive health [21].

The validity and reliability of the Persian version of the scale were confirmed by Foroughan et al. (2008), with specificity and sensitivity at 84% and 90%, respectively [21]. In the present study, the internal consistency of the questionnaire was assessed by Cronbach's α coefficient. (α = 0.86).

The researcher individually completed questionnaires using face-to-face interviews, requiring 30–35 min for each participant.

### Statistical analysis

To analyze univariate data, independent t-tests, ANOVA, and Pearson's correlation coefficient were used, and multiple linear regression was used to investigate the impact of demographic characteristics and lifestyle on cognitive function. All analyses were carried out using SPSS, version 22 (Armonk, NY: IBM Corp.). A p value less than 0.05 was considered significant.

## Results

### Demographic characteristics and cognitive function

In this study, 300 older adults were participated. Of these, 156 older men (52%) and 144 older women (48%) participated. The mean age of the participants was 69.56 ± 7.63 years. The majority of the participants, (60.7%) were in the age group of 60–74 years, while the remaining belonged to the 75–89 age group. Moreover, 134 individuals (44.7%) possessed an educational level below a diploma, 117 individuals (39%) held a diploma, and 49 individuals (16.3%) had attained a university education. 67% of respondents were married, while 99 individuals (33%) comprised various other marital statuses, such as being single, widowed, or having a deceased spouse. One hundred thirty-eight participants (46%) had no recorded history of underlying diseases, whereas 162 participants (54%) were afflicted with underlying health conditions. The mean cognitive function score for the older adults was 22.49 ± 5.44 out of a total score of 30.

The mean cognitive function scores of women aged 60–74 years, married older adults with family cohabitation, and those without underlying diseases were found to be significantly higher than their counterparts (*P* < *0.001*). Additionally, the mean cognitive function scores of the older adults with a standard BMI, possessing a diploma, and holding higher education degrees were significantly higher when compared to other demographic groups (*P* < *0.001*) (Table 1).

**Table 1:** Comparison of the Mean Score of Cognitive Function Among Older Adults Based on Demographic characteristics

Variable		Cognitive function (Mean ± SD)	Statistic	p-value
Gender	Female	23.13 ± 5.31	t* = 1.99	< 0.001
	Male	21.89 ± 5.51		
Age range (year)	60–74	26.43 ± 2.68	t = 35.84	< 0.001
	75–89	16.40 ± 1.76		
Marital status	Married	23.59 ± 4.91	t = 9.41	< 0.001
	Other (single, widow, and deceased spouse)	22.42 ± 2.16		
BMI	Normal	24.45 ± 5.01	t = 9.97	< 0.001
	Overweight	18.72 ± 4.10		
Underlying disease	No	26.95 ± 2.34	t = 20.07	< 0.001
	Yes	18.68 ± 4.32		
Level of education	Below diploma	16.84 ± 2.16	F** = 1041.55	< 0.001
	Diploma	26.73 ± 1.84		
	University degree	27.79 ± 1.35		
Cohabitation with family	Yes	23.75 ± 5.19	t = − 7.29	< 0.001
	No	18.94 ± 4.51		
Driving	Yes	27.39 ± 1.45	t = − 14.56	< 0.001
	No	19.96 ± 5.00		
History of heart disease	Yes	16.01 ± 1.02	t = 15.29	< 0.001
	No	24.49 ± 4.63		
Diabetes	Yes	19.66 ± 4.41	t = 5.26	< 0.001
	No	23.38 ± 5.44		
Hypertension	Yes	19.74 ± 5.05	t = 13.26	< 0.001
	No	26.49 ± 2.95		
Hypercholesterolemia	Yes	18.25 ± 4.06	t = 8.41	< 0.001
	No	23.82 ± 5.13		
Other diseases	Yes	17.73 ± 3.74	t = 9.17	< 0.001
	No	23.85 ± 5.07		

*SD* standard deviation, * Independent t-test, ** ANOVA

### The mean score of lifestyles and its dimensions

The mean and standard deviation of the overall lifestyle score of the participants were equal to 72.52 ± 1.0. Highest score was in avoidance of medication dimension (2.44 ± 0.41) and lowest score was in exercise dimension (1.05 ± 0.73). Additionally, mean scores were recorded for physical health (1.45 ± 0.69), weight control and nutrition (1.42 ± 0.48), disease prevention (1.97 ± 0.44), psychological health (1.56 ± 0.43), spirituality (2.06 ± 0.55), social health (1.79 ± 0.56), accident prevention (1.66 ± 0.87), and environmental health (0.78 ± 0.69). 45.3% had a desired lifestyle, 48.7% had an average lifestyle, and 6% had an undesired lifestyle.

The correlation between the mean score of cognitive function and lifestyle as well as its dimensions ranged from 67 to 83%, indicating a positive and significant correlation between the above-mentioned variables. In other words, an increase in the mean of cognitive function resulted in a corresponding increase in the mean of lifestyle and its dimensions, and vice versa (*P* < *0.001*) (Table 2).

**Table 2:** Comparison of the Correlation between the Mean of Cognitive Function, Lifestyle, and Its Dimensions

Dimensions	Physical health	Exercise and wellness	Weight control and nutrition	Disease prevention	Psychological health	Spirituality	Social health	Avoidance of medication	Accident prevention	Environmental Health	Lifestyle
cognitive function	0.81	0.83	0.79	0.82	0.79	0.72	0.72	0.67	0.78	0.78	**0.8**
p-value	< 0.001	< 0.001	< 0.001	< 0.001	< 0.001	< 0.001	< 0.001	< 0.001	< 0.001	< 0.001	** < 0.001**

In order to investigate the effect of demographic characteristics on cognitive function, the results of the multiple regression model showed that assuming the effect of other variables to be constant, the level of education had a significant positive effect on cognitive function. The mean score of cognitive function of the older adults aged 75–89 years was also 3.79 lower compared to the older adults aged 60–74 years, which was statistically significant (P < 0.001) (Table 3).

**Table 3:** Modeling the effect of demographic characteristics on cognitive function using a multiple regression model

Variable	Β^a^	SE^b^	t^c^	P^d^
Gender (male)	0.26	0.26	1.01	0.315
Age group (75–89 years)	− 3.79	0.39	− 9.69	< 0.001
BMI(overweight)	− 0.34	0.25	− 1.35	0.179
Marital status (married)	− 0.27	0.38	− 0.72	0.472
Level of education	2.75	.67	4.08	< 0.001
Income	− 1.06	0.660	− 2.44	0.015
Housing	0.02	0.28	0.06	0.951
Family support	− 0.01	0.26	− 0.05	0.961
Underlying disease	− 0.02	0.33	0.06	0.951
Driving	− 0.22	1.16	− 0.19	0.85
The total score of lifestyle	3.49	0.51	6.84	< 0.001

^a^Beta coefficient in regression

^b^Standard Error

^c^t statistics

^d^P-value

Multiple linear regression analysis was used to predict the effect of lifestyle variables on cognitive function. The findings revealed that in terms of standardized beta coefficients, physical health, environmental health, exercise, accident prevention, and avoidance of medication exerted the most significant positive effect on cognitive function. Conversely, social health exhibited a notable negative influence on cognitive function.

In the multiple regression model with the adjustment of demographic variables, it was observed that physical health, accident prevention, and avoidance of medication retained robust positive effects on cognitive function, as indicated by standardized β. concurrently, social health maintained a significant negative impact on cognitive function.

According to the univariate linear regression, the mean cognitive function score increased by 9.49.

After adjustment for demographic variables, A one unit increase in the lifestyle score (*P* < *0.001*) demonstrated a positive impact on cognitive function. Specifically, for each one-unit increment in the lifestyle score, there was a notable average increase of 3.49 in the cognitive function score, which remained statistically significant (*P* < *0.001*) (refer to Table 4). As evidenced by Adj R^2^ = 0.92, the model elucidated that approximately 92% of the response variations could be explained.

**Table 4:** Effect of Lifestyle Dimensions on Cognitive Function Based on Multiple Regression Model

Variable	Β^a^	SE^b^	S. β^c^	t^d^	P^e^
Physical health	3.03	0.63	0.39	4.81	< 0.001
Exercise	1.97	0.51	0.27	3.88	< 0.001
Weight control and nutrition	− 0.49	0.66	− 0.04	-0.73	0.464
Disease prevention	− 0.15	0.74	− 0.01	− 0.20	0.840
psychological health	− 0.41	0.760	− 0.03	− 0.54	0.592
Spirituality	− 0.66	0.50	− 0.07	− 1.33	0.186
Social health	− 1.84	0.54	− 0.19	− 3.41	0.001
Avoidance of medication	1.20	0.46	0.090	2.61	0.001
Prevention of accidents	1.62	0.44	0.260	3.70	< 0.001
Environmental Health	2.37	0.54	0.30	4.39	< 0.001

^a^Beta coefficient in regression

^b^Standard Error

^c^Standardized beta coefficient in regression

^d^t statistics

^e^P-value

R^2^ = 0.92 (The coefficient of determination in regression)

## Discussion

The present study evaluated the relationship between lifestyle factors and cognitive function among community-dwelling older people. The main findings were as follows: (1) participants had mild cognitive dysfunction (2) Cognitive performance was better in women, younger and married older adults, individuals without underlying diseases, as well as those living with family compared to others, (3) Older adults with normal body mass index, higher education, and higher income levels had better cognitive performance compared to others, (4) age and education level are predictors of cognitive function,(5) participants had an average level of lifestyle, (6) Among the dimensions of lifestyle, the highest mean score was in the dimension of avoiding drugs, while the lowest was in the dimension of exercise. (The best performance was in the domain of avoiding drugs, and the weakest performance was related to the exercise domain.), (7) There was a positive correlation between cognitive performance and lifestyle, as well as its dimensions, (8) A 1-unit increase in lifestyle total score was associated with 9.49 units greater gain in cognitive function, (9) In order, physical health, environmental health, exercise, accident prevention, and avoiding drugs had the greatest positive impact on cognitive performance, while social health had a negative impact on cognitive performance, (10) Dimensions of physical health, prevention of accidents, and avoiding medications can be predictors of cognitive performance.

Our study showed that participants had mild cognitive dysfunction. Based on the available evidence the prevalence of mild cognitive impairment increases with age and can be a precursor to Alzheimer’s disease and other dementia disorders [22, 23]. So, Early diagnosis is crucial, representing secondary prevention against severe cognitive dysfunction [22]. We also examined the influence of demographic variables on cognitive performance, including age, sex, marriage and cohabitation status, education, body mass index and underlying diseases.

As expected, increasing age was associated with cognitive decline. The 75–89 age group exhibited lower cognitive function than the 60–74 age group, In addition, age was a predictor of cognitive impairment,which is consistent with Kushkestani et al.’s findings [24]. The finding may be attributed to that increasing age leads to an increase in diseases, such as cardiovascular disease and metabolic syndrome, and accordingly, increase the risk of cognitive disorders [25]. Actually, Coronary artery disease risk factors such as diabetes, smoking, and the metabolic syndrome are also potentially modifiable risk factors for developing cognitive decline [26].In addition, with increasing age, neurotransmitters, gray matter volume and neocortical synapses decrease, which leads to cognitive impairment [27].

We further found that higher education correlate with better cognitive function, aligning with Renteria et al. and Kushkestani et al.’s Multiple linear regression analysis identifies education level as predictors of cognitive function. Usually, older adults with higher education are more interested in participating in learning processes and cognitive activities. Therefore, challenging the mind positively affects cognitive function and the prevention of dementia [28].In addition Cognitive activity has the potential to enhance cognitive reserve, such as neural plasticity, by involving complex thinking and mental training [29] results [24, 28]. Furthermore, people with higher income had better cognitive performance. Since more years of education are associated with higher income, the higher cognitive performance in people with higher income can be explain.

The cognitive performance of married people was better than others. The findings are in line with previous research revealed that individuals who are divorced are twice as likely as those who are married to experience cognitive dysfunction [30]. According to recent research, marriage and cohabitation may offer crucial protection against cognitive decline through cognitive reserve. From a cognitive reserve perspective, living in a relationship can directly enhance cognitive function by mental and social stimulation [31]. Besides, the female exhibited better cognitive function than the male.one explanation for these findings may be the protective role of female gender against cognitive decline. Female gender has a protective role against cognitive decline due to hormonal status [32].

In parallel with existing studies [33], the present study also demonstrated that overweight and obese participants showed lower levels of cognitive function. It has been suggested that older people with higher BMI have more disabilities and physical limitations [34]. On the other hand, some studies have shown contradictory results [35, 36]. A study by Tolppanen et al. [20] found that older individuals with higher BMI were less likely to develop dementia than those with lower BMI. Based on previous studies, this difference could be due to the effect of BMI on cognitive performance according to underlying health conditions and non-modifiable factors (such as age and sex) [37, 38].

Moreover, the study also indicated that the population's lifestyle is average, aligning with Ajam et al.'s findings [22]. However, Samadi et al. reported an undesirable lifestyle among older adults. At the same time, other studies presented varied results, indicating the influence of factors such as age groups, tools for measuring lifestyle, culture, race and ethnicity, education, income level, and social conditions across different regions. Lifestyle dimension comparisons highlight the lowest physical activity and exercise performance, consistent with Ajam et al.’s study [23]. Other research also underscores suboptimal physical activity levels among older individuals [39], which may be due to physical and economic challenges, limited access to sports facilities, and inadequate support programs for participating in sports programs.

This study investigated the relationship between lifestyle and cognitive function. Healthy lifestyle scores were associated with better cognitive function. Our results were consistent with previous studies [40–42].The underlying mechanisms linking lifestyle factors to cognitive performance are not fully comprehended. The involvement of vascular, inflammatory, neurotoxic, oxidative stress, and psychosocial processes has been proposed by various hypotheses [43]. Lifestyle domains, including physical health, environmental health, exercise, prevention of accidents, and avoidance of drugs, demonstrate the most positive effects on cognitive performance. Numerous observational studies have reported protective effects of exercise and physical activity against age-related cognitive decline. [44, 45]. In addition, Interventional studies found an increase in hippocampal size and perfusion following exercise intervention [46, 47]These effects might also be due to neuronal and vascular adaptations that enhance cognition through neurogenesis, angiogenesis, synaptic plasticity, reduction in proinflammatory processes, and mitigation of cellular damage from oxidative stress. [48].

The current study indicates that physical health, prevention of accidents, and avoidance of drugs and harmful substances predict cognitive function. It can be said that a healthy lifestyle enhances physical performance and health, positively influencing mental health and cognitive function [19, 49].

An interesting finding in our study was the inverse relationship between social health and cognitive performance, encompassing people's social skills and performance [50]. Consistent with the present study, the study results of Lam et al. showed that increasing social activity increased the risk of cognitive disorders. One possible explanation is that the long-term performance of a simple and monotonous social activity has no positive impact on cognitive function [51]. This finding is contrary to many existing studies [52, 53].The present inconsistency may result from unexplored variables (e.g., personality traits of older adults) that were not addressed in this study and should be further investigated in other studies.

This study has limitations that need to be noted. First, the causal relationship is still uncertain because a cross-sectional study may not fully assess the temporality between lifestyle and cognitive outcome. To address the limitation, we need a long-term follow-up study. Second, the population was selected from the comprehensive urban health centers of Khorram Abad, limiting generalizability to older adults in rural areas. Therefore, conducting research in rural communities is also suggested.

## Conclusion

This study highlighted the association between cognitive function and lifestyle. Older individual who had an unhealthy lifestyle had poorer cognitive performance. Physical activity had lower score among older adults. Age and education were predictors of cognitive performance. Additionally, among lifestyle dimension physical health, prevention of accidents, and avoidance of drug were predictors of cognitive function.

Given that lifestyle behavior is a self-regulating and relatively easy target for preventing cognitive decline, lifestyle modification could be a cost-effective prevention strategy, particularly in low- and middle-income countries that lack health-care resources. Therefore, Strategic planning for a healthier lifestyle for older adults, especially in exercise and physical activity needs to be prioritized.

## Data Availability

Datasets are available upon reasonable request from the corresponding author.

## Abbreviations

BMI: Body mass index
MMSE: Mini-Mental State Examination
